# Short-Term Tomato Consumption Alters the Pig Gut Microbiome toward a More Favorable Profile

**DOI:** 10.1128/spectrum.02506-22

**Published:** 2022-11-08

**Authors:** Mallory L. Goggans, Emma A. Bilbrey, Cristian D. Quiroz-Moreno, David M. Francis, Sheila K. Jacobi, Jasna Kovac, Jessica L. Cooperstone

**Affiliations:** a Food Science and Technology, The Ohio State University, Columbus, Ohio, USA; b Horticulture and Crop Science, The Ohio State University, Columbus, Ohio, USA; c Horticulture and Crop Science, The Ohio State University, Wooster, Ohio, USA; d Animal Sciences, The Ohio State University, Columbus, Ohio, USA; e Food Science, The Pennsylvania State University, University Park, Pennsylvania, USA; f Microbiome Center, Huck Institutes of the Life Sciences, The Pennsylvania State University, University Park, Pennsylvania, USA; Royal Agricultural University

**Keywords:** gut microbiome, pig, tomato, nutrition

## Abstract

Diets rich in fruits and vegetables have been shown to exert positive effects on the gut microbiome. However, little is known about the specific effect of individual fruits or vegetables on gut microbe profiles. This study aims to elucidate the effects of tomato consumption on the gut microbiome, as tomatoes account for 22% of vegetable consumption in Western diets, and their consumption has been associated with positive health outcomes. Using piglets as a physiologically relevant model of human metabolism, 20 animals were assigned to either a control or a tomato powder-supplemented diet (both macronutrient matched and isocaloric) for 14 days. The microbiome was sampled rectally at three time points: day 0 (baseline), day 7 (midpoint), and day 14 (end of study). DNA was sequenced using shotgun metagenomics, and reads were annotated using MG-RAST. There were no differences in body weight or feed intake between our two treatment groups. There was a microbial shift which included a higher ratio of *Bacteroidota* to *Bacillota* (formerly known as *Bacteroidetes* and *Firmicutes*, respectively) and higher alpha-diversity in tomato-fed animals, indicating a shift to a more desirable phenotype. Analyses at both the phylum and genus levels showed global microbiome profile changes (permutational multivariate analysis of variance [PERMANOVA], *P* ≤ 0.05) over time but not with tomato consumption. These data suggest that short-term tomato consumption can beneficially influence the gut microbial profile, warranting further investigation in humans.

**IMPORTANCE** The composition of the microorganisms in the gut is a contributor to overall health, prompting the development of strategies to alter the microbiome composition. Studies have investigated the role of the diet on the microbiome, as it is a major modifiable risk factor contributing to health; however, little is known about the causal effects of consumption of specific foods on the gut microbiota. A more complete understanding of how individual foods impact the microbiome will enable more evidence-based dietary recommendations for long-term health. Tomatoes are of interest as the most consumed nonstarchy vegetable and a common source of nutrients and phytochemicals across the world. This study aimed to elucidate the effect of short-term tomato consumption on the microbiome, using piglets as a physiologically relevant model to humans. We found that tomato consumption can positively affect the gut microbial profile, which warrants further investigation in humans.

## INTRODUCTION

Research has shown that the composition of the gut microbiome can be an effector of overall health (1). The composition of these gut microorganisms has been associated with a number of chronic diseases, such as cardiovascular disease (2), inflammation (3), type 2 diabetes (1), and obesity (3–5). As diet is a major modifiable factor of health, there is interest in elucidating how dietary factors can alter the microbiome (6, 7). While it is possible to use some microbiome endpoints and associate them with health (i.e., a more diverse community is favorable [1, 6, 8], as is a lower ratio of *Bacteroidota* to *Bacillota* [formerly known as *Bacteroidetes* and *Firmicutes*, respectively] [4]), the reality is that bias in sequencing approaches as well as differences in microbial communities due to lifestyle factors and location adds complexity to this interpretation (9). Still, diets rich in fruits, vegetables, and whole grains have been consistently associated with a healthier microbiome (6–8, 10). However, discerning the way specific foods might affect the microbiome using intervention studies remains largely uninvestigated. Understanding the global effects that specific foods have on the microbiome helps contextualize the effect they are having toward overall health and sets a foundation toward making personalized nutritional recommendations.

Tomatoes are of interest as one such specific food because they are a common source of nutrients for many around the world. They are the second most commonly consumed vegetable (11) and are an important specialty crop across the United States. Over 12 million metric tons of tomatoes is produced in the United States each year (12), with Americans consuming about 30 pounds per person in 2018 (13). Tomatoes are a rich source of essential nutrients (e.g., vitamins A and C), fiber, and phytochemicals (e.g., lycopene, flavonoids, and phenolic acids). Tomato consumption has been linked to protection against various chronic diseases (14–16), though causality about the mechanism of action is not well understood.

We hypothesized that one mechanism by which tomatoes provide a health benefit is through their modulation of the gut microbiome. Preliminary microbiome studies in mice, feeding them tomatoes or their phytochemicals, have shown positive outcomes, including increased microbial diversity, decreased abundance of *Clostridium* spp., and decreased symptoms of irritable bowel disease (17–21). Here, we aimed to elucidate the effects of short-term, consistent tomato consumption on the gut microbial ecosystem, using pigs as a physiologically relevant model (22–24) for humans. To investigate this question, we fed weaned piglets (*n* = 20, aged 4 weeks) a diet supplemented with 10% (wt/wt) tomato powder or an isocaloric and macronutrient-matched control diet for 2 weeks, sampling the gut microbiome via rectal swab at three points during the experimental period. The use of macronutrient-matched diets allowed us to test the effect of tomato phytochemicals on the microbiome of studied pigs, rather than the effect of differences in nutrients, such as fiber or sugar. DNA from rectal swabs was subjected to shotgun metagenomic sequencing (i.e., the untargeted sequencing of all the DNA present in a sample [25]). The resulting reads were annotated and analyzed at both the phylum and genus levels using univariate and multivariate approaches, including the analysis of beta-diversity, relative abundances of *Bacteroidota* and *Bacillota* and their ratio, and alpha-diversity.

## RESULTS AND DISCUSSION

### Diet type did not affect animal weight.

**FIG 1:**
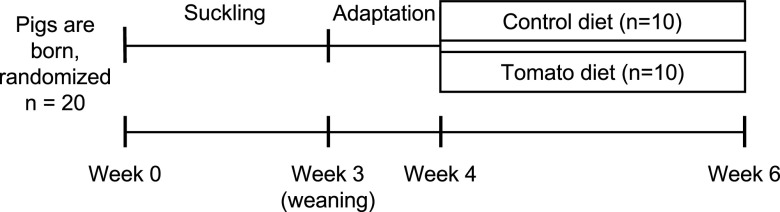
Overall animal study design. Pigs were adapted to a dry diet from weeks 3 to 4. Microbiome was sampled via rectal swabs when pigs were aged 4 weeks (day 0, baseline), 5 weeks (day 7, midpoint), and 6 weeks (day 14, end of study) for shotgun metagenomics.

An overall scheme of the animal study design can be found in Fig. 1. Pigs were weighed and feed intake was measured weekly. There was no difference in feed intake or animal weight by diet at any time point over the trial (see Table S1 in the supplemental material). Health of the pigs was not altered by dietary treatment.

### A median sequence depth was 2.5M reads.

Each sample’s forward and reverse reads were checked for quality using FastQC version 0.11.9 (26). All sequence files passed quality checks, and no samples had to be discarded. Thirteen of the 60 total samples were resequenced to a median of 2.3 million (2.3M) reads per sample. For resequenced samples, sequences from the first and second sequencing run were merged, checked for quality, and used for further analyses. Rarefaction curves demonstrate that a similar species richness was achieved in samples with differing sequence depths (Fig. S1). A recent study has shown that even shallow shotgun metagenomics (<500K reads/sample) provides better annotation of taxonomic and functional composition of microbiome than does 16S rRNA sequencing (27), providing our rationale for this sequencing approach.

### *Bacillota* (i.e., *Firmicutes*) were the predominant phylum and *Prevotella* the most abundant genus detected in the pig fecal microbiome.

The mammalian gut microbiome is a complex microbial ecosystem; hence, it is beneficial to conduct analyses at more than one taxonomic rank, as the profile of each rank provides different types of information. The average human gut microbiome is dominated by *Bacteroidota* (formerly known as *Bacteroidetes*) and *Bacillota* (formerly known as *Firmicutes*), which typically account for 70 to 90% of the total microbiome makeup (1). Analyses of phyla often reveal changes in the proportions of the dominant few, thus providing a broad picture of the state of the gut microbiome. Alternatively, genera are highly diverse, often with hundreds of taxa identified (28). These analyses provide a finer resolution of microbiome composition. Here, we aimed to capture modifications of the microbiome at both the phylum and genus level. For this reason, all analyses (aside from those specific to phyla) were completed at both taxonomic ranks.

Across all pigs, annotation using MG-RAST and filtering for data quality resulted in identification of 45 phyla. Of those, 28 were from the domain *Bacteria*, comprising on average 99.3% ± 0.2% of the total reads, 10 were from *Eukaryota*, 5 were *Archaea*, 1 was a virus, and 1 was unclassified. The most prevalent phyla were *Bacillota* (formerly known as *Firmicutes*; 52.7% average abundance ± 5.5% standard deviation), *Bacteroidota* (formerly known as *Bacteroidetes*, 35.4% ± 5.9%), *Actinomycetota* (formerly known as *Actinobacteria*) (4.7% ± 1.8%), *Pseudomonadota* (formerly known as *Proteobacteria*) (3.9% ± 1.2%), and *Fusobacteriota* (formerly known as *Fusobacteria*) (0.43 × 10^−4^% ± 8.5 × 10^−4^%). Similar relative abundances of phyla were observed across samples, regardless of the diet groups. Previous studies reported conflicting results in terms of predominant phyla in pig microbiome. Some studies have shown *Firmicutes* to be the most abundant phylum in the pig gut microbiome after weaning (29, 30), while others have reported *Bacteroidetes* as the dominant phylum (31).

Annotation from MG-RAST and filtering for data quality resulted in the identification of 755 genera. Of these 755 genera, 582 were in the *Bacteria* domain, 89 were *Eukaryota*, 60 were *Archaea*, 23 were viruses, and 1 was unclassified. Overall, the most prevalent genera were *Prevotella* (22.23% average abundance ± 5.4% standard deviation), *Bacteroides* (10.34% ± 1.9%), *Clostridium* (8.56% ± 1.8%), *Lactobacillus* (6.78% ± 4.6%), and *Eubacterium* (5.16% ± 1.0%). These genera were detected in similar relative abundances in each group when data were parsed by diet. Previous reports have shown *Prevotella*, *Bacteroides*, and *Clostridium* to be the most abundant genera in pig gut microbiomes (30), which is consistent with our findings.

### Beta-diversity changed over time but was not significantly affected by the tomato-supplemented diet.

**FIG 2:**
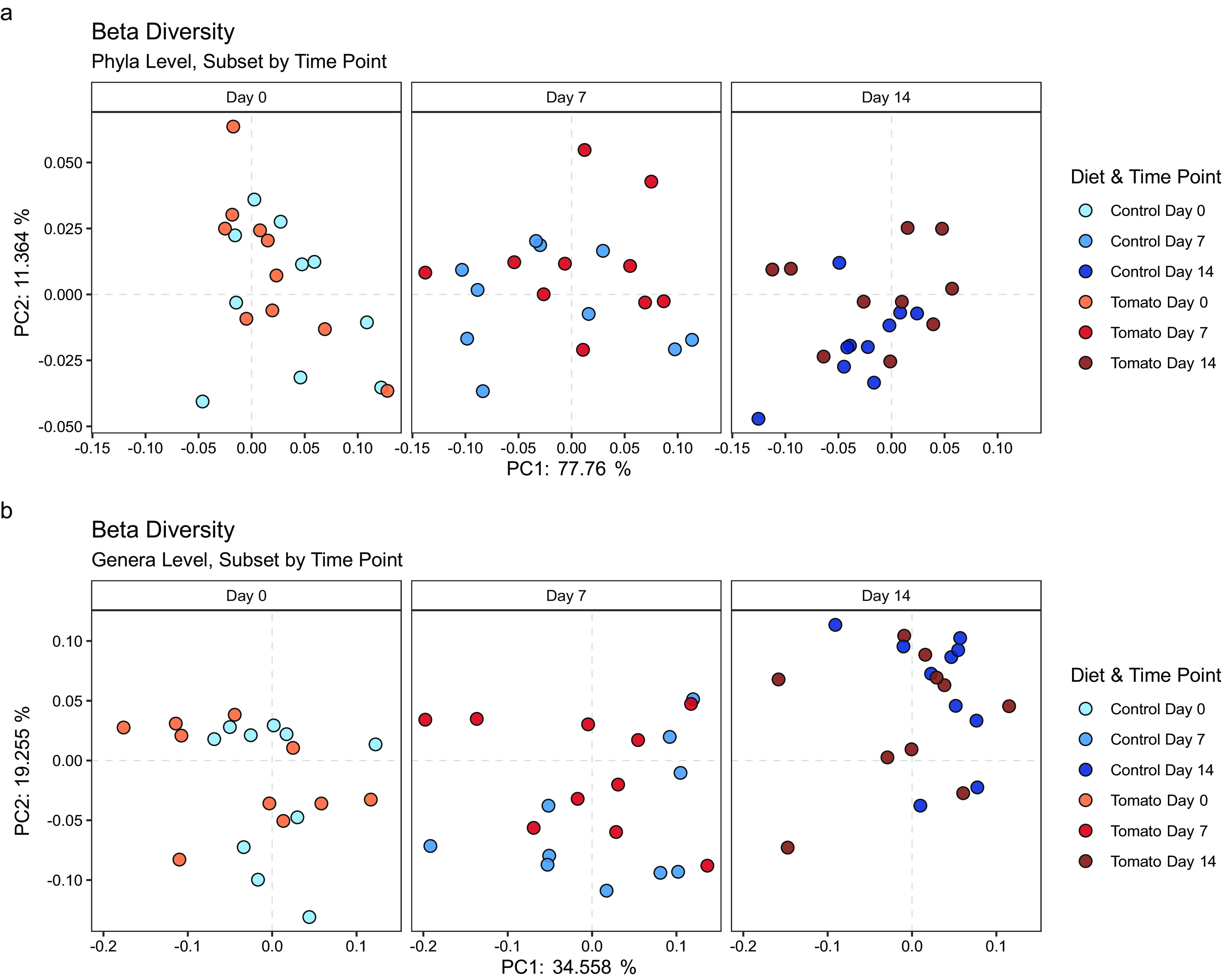
Principal-coordinate analysis (PCoA) using Bray-Curtis distances showing beta-diversity of the whole microbiome at the phylum (a) level and genus (b) level. Each dot represents a sample collected from one pig. Plots are faceted by diet. Using repeated-measures PERMANOVA (model: beta-diversity = diet + time point + diet × time point + error), only a significant effect of time point was detected at both the phylum (*P* = 0.020) and genus (*P* = 0.005) levels.

To understand the beta-diversity (differences between the microbial communities) of pigs on different diets and at different time points, all data were first visualized via principal-coordinate analysis (PCoA) using the Bray-Curtis dissimilarity metric. PCoA plots (Fig. 2) were created for both phyla and genera separately using the relative abundances of all samples. Plots were faceted by diet to observe sample clustering by time point more easily. PC1 and PC2 together accounted for 89.1% of the variation in the phylum-level microbiome and 53.8% at the genus level. Visual clustering in PCoA score plots at either taxonomic level was not easily observed between diets, but within the control diet, grouping was observed according to time point. It is not surprising that overall microbiome profile differences are not evident in the PCoA plots due to presence or absence of a single component of a diet (i.e., tomatoes). Global differences in microbiome composition are more likely to be observed when two completely different diets are fed, as previously shown when comparing the effects of a plant-based diet and animal-based diet on the microbiome (32).

In order to determine the significance of observed trends in the PCoA due to diet and time point, permutational multivariate analysis of variance (PERMANOVA) was used (model: beta-diversity = diet + time point + diet × time point + error, where each pig was a plot containing three samples collected over time). These multivariate-restricted permutation tests are a useful approach for assessing differences in beta-diversity because they allow for the investigation of the gut microbiome as a whole, instead of focusing on individual taxa. The PERMANOVA model *P* values are recorded in Table 1. At both the phylum and genus levels, we found the interaction term to be nonsignificant (phylum, *P* = 0.510; genus, *P* = 0.360), and therefore, we removed it from the model. The new model was then tested and revealed an overall significant effect of time point (phylum, *P* = 0.020; genus, *P* = 0.005) but not diet (*P* = 0.270) on the gut microbiome (Table 1).

**TABLE 1:** Results from restricted permutation tests via PERMANOVA to investigate differences in beta-diversity at the phylum and genus taxonomic levels^*c*^

Variable	Phylum	Genus
Diet	0.270	0.060
Time point	0.020^*a*^	0.005^*b*^
Diet × time point	0.510	0.360

a Indicates significant model effect at a *P* value of ≤0.05.

b Indicates significant model effect at a *P* value of ≤0.01.

c The full model tested the variance explained by the diet, time point, and their interaction on the dissimilarity matrix, calculated with Bray-Curtis distances.

These data can be interpreted as that, at both taxonomic ranks, the microbiomes of pigs were significantly changing over the 2-week intervention, but the effect of diet on beta-diversity was not significant. In another study using a mouse model, the microbiomes were compared between a group fed a high-fat diet supplemented with tomato powder and a high-fat-only diet group. Using clustering by unweighted UniFrac dissimilarity, a significant difference was detected between diet group microbiomes (17). However, using weighted UniFrac distances, no separation of tomato and control groups was observed in the pigs. It is possible that using a dissimilarity measure that incorporates evolutionary relatedness may have been a contributor to the detected significant effects. However, a direct comparison with our study is difficult because mice are known to be different than pigs in their microbiome composition (33).

### Inverse relationship between *Bacteroidota* and *Bacillota* abundances was detected over time in the control-fed pigs but not tomato-fed pigs.

**FIG 3:**
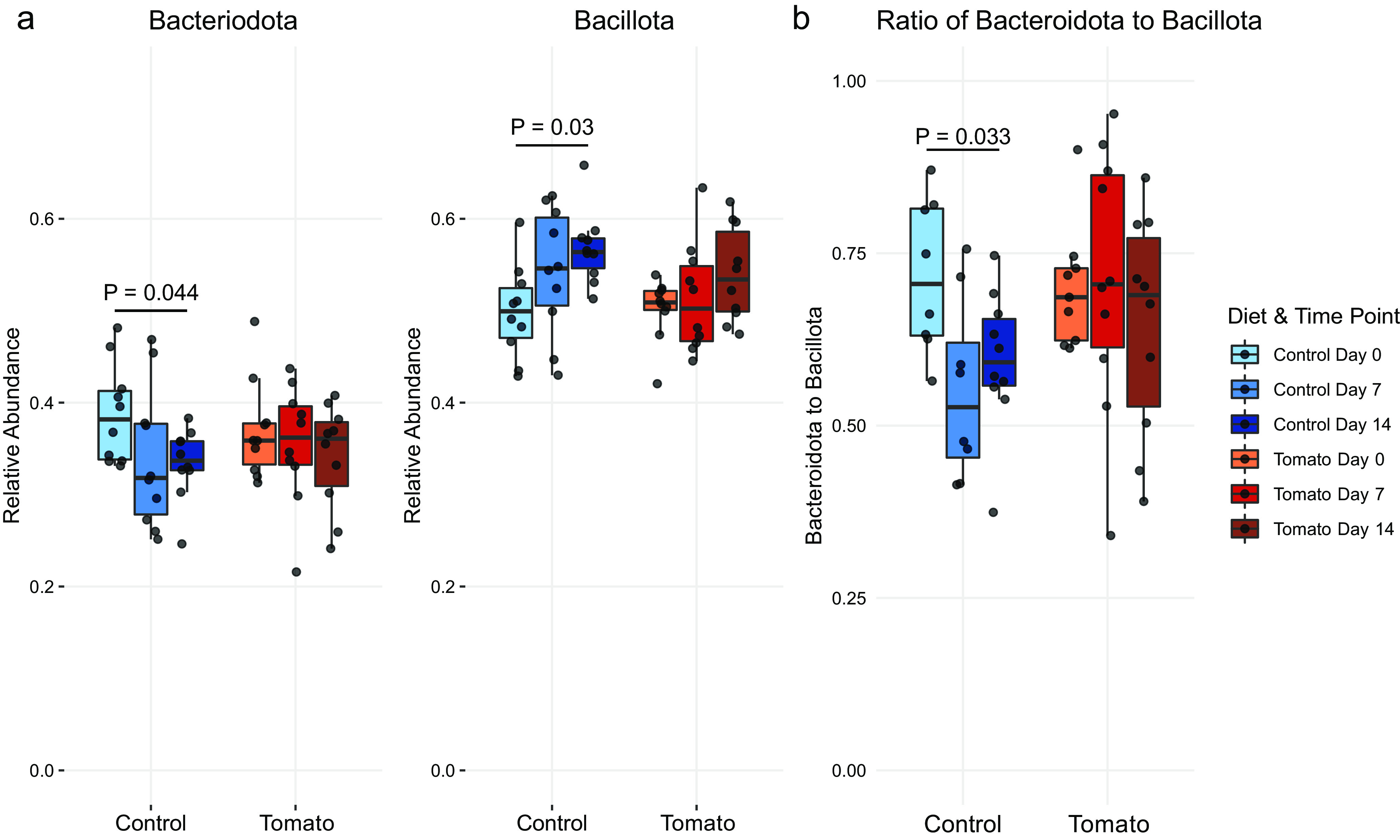
(a) Comparing relative abundances across time points and between diets for two phyla, *Bacteroidota* and *Bacillota*. Using repeated-measures ANOVA, a significant model effect of time point was found for both phyla (*Bacteroidota*, *P* = 0.024; *Bacillota*, *P* = 0.001). *Post hoc* findings of significant differences between day 0 control and day 14 control were found for both *Bacteroidota* (*P* = 0.044) and *Bacillota* (*P* = 0.03). No significant effects of diet or time point-by-diet interaction were detected for either phylum. (b) Comparing the ratios of the relative abundance of *Bacteroidota* to that of *Bacillota* across time points and between diets. A significant effect of time via repeated-measures ANOVA (*P* = 0.009) led to *post hoc* comparisons and a significant difference in the ratio of *Bacteroidota* to *Bacillota* between day 0 and day 14 in control-fed pigs only (*P* = 0.033). There was no significant effect of diet or time point-by-diet interaction.

In addition to multivariate approaches to understand microbiome data, univariate methods to examine differences in specific taxa are valuable. As previously stated, the phyla *Bacteroidota* (i.e., *Bacteroidetes*) and *Bacillota* (i.e., *Firmicutes*) and their relationship have been implicated in obesity and high-fat diets (34, 35). With these *a priori* interests, changes in these two phyla were assessed individually across diets and time points using repeated-measures ANOVA. Results indicated a significant model effect of time point for both phyla (*Bacteroidota*, *P* = 0.024; *Bacillota*, *P* = 0.001), whereas diet and the interaction term were nonsignificant. After *post hoc* analyses to determine which pairwise groups differed, significant alteration in the abundance of both *Bacteroidota* and *Bacillota* was found between day 0 and day 14 in control-fed pigs (*Bacteroidota*, *P* = 0.044; *Bacillota*, *P* = 0.03). No significant differences between time points within the tomato-fed pigs were observed. Box plots of the two phyla demonstrate the inverse relationship between *Bacteroidota* and *Bacillota* abundances over time in the control-fed pigs (Fig. 3a).

Additionally, the ratio of *Bacteroidota* to *Bacillota* in the gut microbiome is a commonly assessed metric because of its correlation with obesity (4, 34, 35). Therefore, differences in *Bacteroidota*/*Bacillota* were also tested via repeated-measures ANOVA with diet, time point, and their interaction as factors. This analysis revealed a significant difference due to time (*P* = 0.009), with a nonsignificant effect of diet (*P* = 0.728) and time-by-diet interaction (*P* = 0.436). *Post hoc* analyses using pairwise comparisons (and adjusting for multiple comparisons using the Benjamini-Hochberg procedure [36]) showed a significant difference only in the control-fed group between day 0 and day 14 (*P* = 0.033) (Fig. 3b). There were no statistically significant changes in *Bacteroidota*/*Bacillota* detected within tomato-fed pigs. The significant *Bacteroidota*/*Bacillota* decrease found in the control-fed group at day 14 versus baseline corresponds with the significant decrease in *Bacteroidota* and increase in *Bacillota* mentioned above.

These data together suggest that incorporation of tomato into the diet can help prevent the alteration of the microbial profile to maintain higher *Bacteroidota*/*Bacillota* ratio. A low *Bacteroidota*/*Bacillota* ratio in the gut has been linked to an obese host (4, 37, 38), suggesting a higher *Bacteroidota*/*Bacillota* ratio is more desirable. In our control pigs, *Bacteroidota*/*Bacillota* decreased over time, whereas the ratio remained unchanged in tomato-fed pigs, so it follows that tomato consumption may be playing a role in maintaining a more desirable *Bacteroidota*/*Bacillota* ratio. It has been suggested that altering this ratio may directly affect risk of obesity, as there is some evidence that taxa in the *Firmicutes* phylum have an increased capacity for energy harvest (5, 39). The role of *Bacteroidota*/*Bacillota* in predicting or influencing obesity and the mechanisms underlying this relationship, including diet, are worth further investigation.

Diet is known to have a major influence on the gut microbiome in general (6, 8), and limited studies showed that certain dietary patterns or components affect *Bacteroidota*/*Bacillota* ratio. Some studies have demonstrated that fiber, starch, and other plant polysaccharides can increase the *Bacteroidota*/*Bacillota* ratio (8, 40, 41). Tomato powder does provide a source of these carbohydrates, although our control diet was macronutrient matched to the tomato diet, suggesting differences we see here are a function of the small-molecule phytochemicals from tomato. Some bacteria are known to metabolize tomato phytochemicals, such as rutin, quercetin, and chlorogenic acid (7, 42). Adding a food with unique phytochemicals to the diet introduces a new source of nutrients for the microbiome and encourages growth of certain bacteria, suggesting a mechanism in which phytochemicals indirectly influence the makeup of the microbiome. Effects shown here could be partially or wholly induced by tomato phytochemicals; however, it is also possible that certain polysaccharides in tomatoes provide benefits, preventing the change in *Bacteroidota*, *Bacillota*, and *Bacteroidota*/*Bacillota* ratio seen in the control-fed animals over time. A study that fed tomato powder to mice with induced liver cancer saw a decreased level of *Bacteroidota* and an increased level of *Bacillota*, resulting in a lower *Bacteroidota*/*Bacillota* ratio (18). However, these animals were double knockouts deficient in beta carotene oxygenase 1 and 2, which is known to exert physiological affects beyond metabolism of carotenoids, challenging the translation of these results to other mammals (43, 44).

### Several phyla were detected in significantly higher relative abundance in tomato-fed pigs than in control pigs after 14 days of feeding.

In addition to assessing *Bacteroidota* and *Bacillota*, which were of *a priori* interest, we assessed changes in each of the 45 detected phyla across time points and between diet groups. Differences between relative abundances of individual taxa were determined by compositional analyses using the ALDEx2 package in R (45–48). Within control-fed pigs, there were no significant changes in relative abundance of any phylum over time. While we would expect to see differences due to time in *Bacteroidota* and *Bacillota*, as was discovered with repeated-measures ANOVA, we suspect that due to the multiple testing corrections incurred to test the 45 phyla, this test is conservative in its estimate of changes in taxon relative abundance. Within tomato-fed pigs, 1 phylum (unclassified [*Bacteria* derived]) of the 45 detected was significantly altered over time. When comparing diet groups, there were no significant phylum-level differences at day 0, 1 phylum differed (unclassified [*Bacteria* derived]) on day 7, and 5 phyla (*Nematoda*, *Apicomplexa*, *Deinococcus*-*Thermus*, *Pseudomonadota* [i.e., *Proteobacteria*], and unclassified [*Bacteria* derived]) differed on day 14. The relative abundance of each of these phyla was found to be higher in the tomato-fed group than in the control, apart from *Deinococcus*-*Thermus*, for which the opposite was true. The full list of *P* values for all phylum-level comparisons can be found in Table S5 in the supplemental material.

No significant difference at day 0 is expected, as no intervention had yet occurred, and microbiome compositions should be relatively consistent between pigs. Providing an explanation for the functional implications of changes in phyla at the other two time points is challenging, as most have not been extensively studied in the context of the gut microbiome and each contains diverse genera and species that vary in function.

To get closer to understanding functional implications of differences in taxa across time points and between diet groups, the same compositional analyses were conducted using ALDEx2 at the genus level. Significant differences were detected in relative abundances of 4 genera across time in control-fed pigs. These were *Oribacterium*, Streptococcus, *Lactococcus*, and *Granulicatella*, all of which were detected in a higher relative abundance with time. In tomato-fed pigs compared to control-fed pigs, four genera were found to have significantly increased in relative abundance over time: Staphylococcus, *Alphatorquevirus*, Lambda-like viruses, and an unclassified group (*Bacteria* derived).

In the context of the gut microbiome, changes in *Lactococcus* (phylum *Firmicutes*) and Staphylococcus (phylum *Firmicutes*) abundances are of interest. Some *Lactococcus* species and strains have shown potential to act as a probiotic in the gut and provide some health benefits in animal studies (49, 50). In contrast, this genus has also been associated with body fat accumulation in mice fed a high-fat diet (51). More work is needed to determine its exact role. Here, we report an increase in *Lactococcus* relative abundance over time within the microbiomes of the control-fed pigs, resulting in a significant difference between diet groups at day 14. Many species within the Staphylococcus genus are known to be typical commensal inhabitants of the human and pig skin microbiomes (52, 53). However, there are some species which can cause pathogenesis in humans (54). Without further knowledge of the species present in these samples, it is impossible to say whether increases in Staphylococcus abundance in tomato-fed pigs should be viewed as negative. However, it should be noted that no pigs showed signs of diseases throughout the study.

Furthermore, significant differences were assessed between diet groups for each genus. As in phylum-level analyses, no significant differences in abundance of genera were noted between diet groups at day 0. At day 7, an unclassified group (*Bacteria* derived) was significantly different between diets, consistent with the single phylum (unclassified [*Bacteria* derived]) for which a difference was detected in the phylum-level analyses. Analyses of differences at day 14 showed 14 genera significantly different in relative abundance. These were *Alphatorquevirus*, *Brugia*, *Loa*, *Malassezia*, *Plasmodium*, *Propionibacterium*, *Roseiflexus*, *Saccharomyces*, Staphylococcus, *Stenotrophomonas*, Streptococcus, *Vanderwaltozyma*, Lambda-like viruses, and unclassified (*Bacteria* derived). All were significantly higher in the tomato-fed than in the control group, except for *Roseiflexus* and Streptococcus, which were higher in the control group. There is evidence that *Propionibacterium* is an early colonizer of the infant gut (55), with its enrichment protective against necrotizing enterocolitis (56), and acts as a probiotic (57). Similarly, some *Saccharomyces* species have also been shown to be probiotic, increasing the abundance of *Bacteroidota* and decreasing that of *Bacillota* (58), while others act along the gut-brain axis in reducing irritable bowel disease severity (59). Increased Streptococcus has been associated with increased localized inflammation (60), while other strains have been shown to be probiotic (61). However, it is currently difficult to contextualize these findings because of the diversity of species within each genus. The full list of *P* values for all genus-level comparisons can be found in Table S6.

### Tomato-fed pigs had a significantly higher fecal microbiome alpha-diversity at a phylum level but not at a genus level.

**FIG 4:**
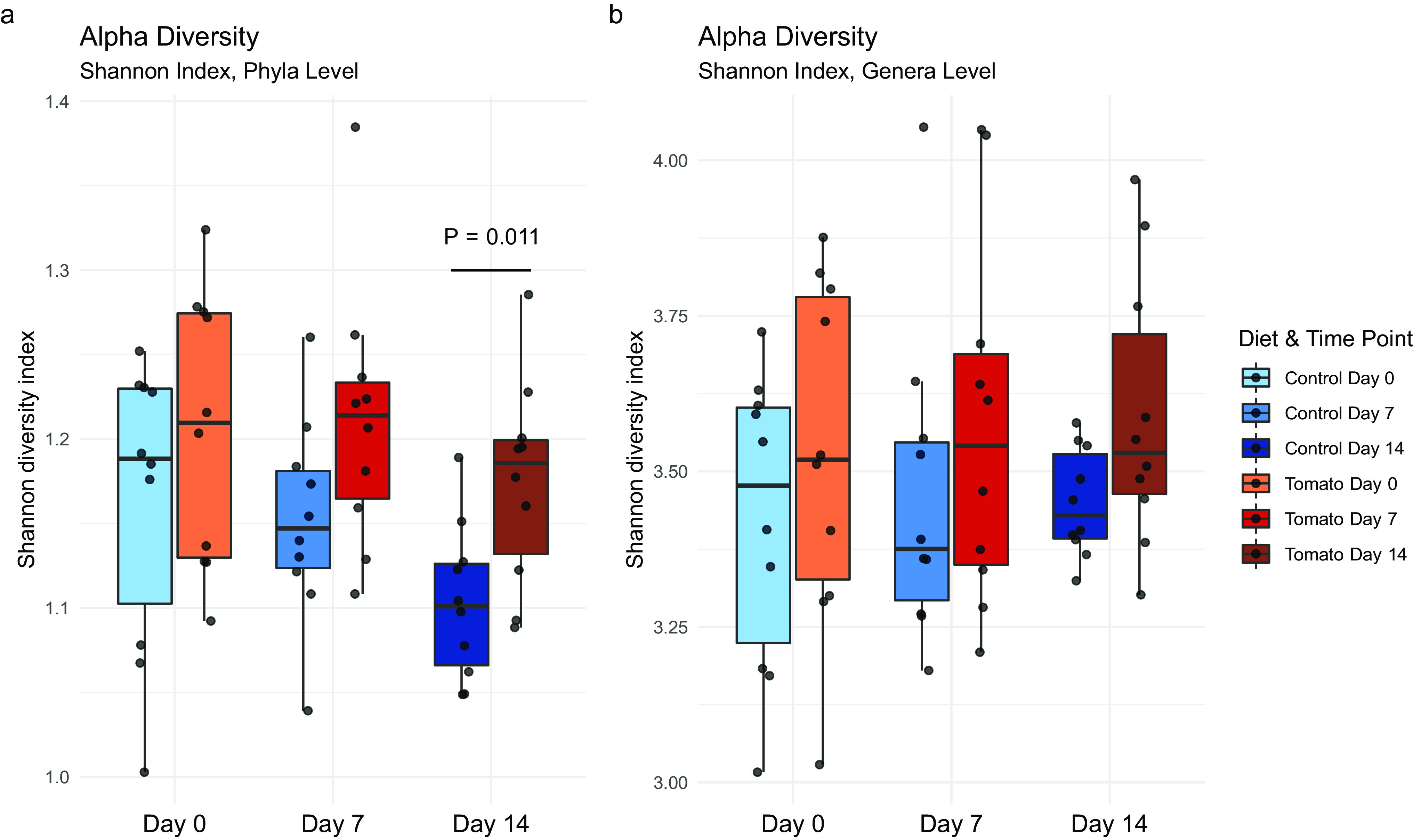
Alpha-diversity as measured by the Shannon diversity index at the phylum (a) and genus (b) level. (a) A statistically significant effect of diet was found via repeated-measures ANOVA (*P* = 0.004), and a *post hoc* difference (*P* = 0.011) was found at the phylum level between control- and tomato-fed pigs at day 14. (b) No significant differences were observed at the genus level.

The microbiome is a complex collection of organisms, so it is important to analyze differences in the community not only on the basis of single phyla and genera but also by examining the overall diversity present. Therefore, using the Shannon index, alpha-diversity was calculated at the phylum and genus level for each sample to provide a measure of taxonomic diversity within each sample. Diet and time point group averages were then compared with a repeated-measures ANOVA (Fig. 4).

Comparison of phylum-level alpha-diversity between diets and time points showed a significant effect of diet on alpha-diversity (*P* = 0.004) but no significant effect of time (*P* = 0.086) or diet-by-time interaction (*P* = 0.791). *Post hoc* analyses by pairwise comparison revealed a statistical difference between control- and tomato-fed pigs at day 14 (*P* = 0.011), with higher alpha-diversity in the tomato-fed animals (Fig. 4a). This aligns with our univariate ALDEx2 analyses, as significant differences in 5 phyla were observed between the diets at day 14. Consumption of tomato has previously been shown to affect alpha-diversity. Mice consuming high-fat diets supplemented with tomato powder had higher levels of alpha-diversity than those who did not consume tomato powder (17, 18). Higher alpha-diversity is desirable, as a more diverse gut microbiome has been associated with more benefits for the host and better resilience to pathogens (28).

The repeated-measures ANOVA investigating the effect of diet, time point, and their interaction on alpha-diversity at the genus level showed no significant differences (Fig. 4b). The lack of observed effect has been similarly noted in human interventions with single foods, including broccoli (62). Another study showed that walnut consumption significantly increased alpha-diversity in rats (63). Again, few studies have been conducted with single plant food interventions for comparison to our results here.

The gut microbiome has a large amount of functional redundancy at the genus and species level, meaning multiple microorganisms contribute the same metabolic functions (28). For example, there are numerous different organisms, when annotated at the genus level, that metabolize carbohydrates, others that metabolize proteins, and some that overlap and metabolize both macromolecules. This provides stability and resiliency to the microbial ecosystem of the gut through a consistent use of nutrients and output of metabolites, even if the exact genus or species presence is changing. Dietary causes of change in alpha-diversity typically occur from repeated habits or patterns that are sustained and dominated by one macronutrient, such as consistent high fat intake, because this limits the available nutrients for microbes (28).

We elected to conduct this experiment in pigs (instead of in mice or rats) because a porcine model has been shown to be more anatomically and metabolically similar to humans (33).

In summary, we have found that supplementation of the diet with 10% tomato powder (compared to a macronutrient-matched control) has the ability to modulate the gut microbiome in pigs. Animals on tomato-containing diets had higher alpha-diversity, a higher *Bacteroidota*/*Bacillota* ratio, higher abundance of *Bacteroidota* (i.e., *Bacteroidetes*), and lower abundance of *Bacillota* (i.e., *Firmicutes*), consistent with a more beneficial microbial phenotype. However, we acknowledge that this is a short-term study with a small number of animals (*n* = 10 per group), and longer and larger studies should be conducted to confirm and expand on the findings we report here. The effect of tomato consumption on the gut microbiome in humans warrants further investigation at a functional level to improve the understanding of the effect of a tomato-rich diet on the functional resilience of the human gut microbiome.

## MATERIALS AND METHODS

### Experimental diet production.

Processing tomatoes (Solanum lycopersicum L.) used in this study were grown at the North Central Agricultural Research Station of Ohio State University (OSU) in Fremont, OH. A hybrid tomato derived from the cross OH8245 × OH8243 (64) was used. Tomatoes were grown using conventional horticultural practices, mechanically harvested using a Guaresci harvester (Guaresci, Sp.A, Pilastri, Italy), and sorted to include ripe fruits only. Tomatoes were transported to the Columbus, OH, campus of OSU and processed at the Food Industries Center Pilot Plant, where fruits were immediately washed, diced, and frozen, as previously described (65). Frozen tomatoes were freeze-dried, and dry material was ground into a fine powder using a vertical chopper mixer (65). Tomato powder was stored in vacuum-sealed bags at −20°C until use.

The basal diet (Table 2) was formulated with a nutrient makeup appropriate for nursery pigs weighing 7 to 11 kg according to the National Research Council (66). To the basal diet, the tomato powder was added at 10% (wt/wt). To create the control diet, the basal diet was supplemented with milk protein isolate (90% purity, 13%, protein), powdered sugar (70%, sugar), pectin (3.4%, soluble fiber), and cellulose (13.6%, insoluble fiber) to create a macronutrient match to the tomato diet (Table 2). These ingredients were formulated to match the ratios of nutrients typically found in tomato powder as reported by Food Data Central (67). This supplement was added at 10% (wt/wt) to match the addition of the tomato powder.

**TABLE 2:** Composition of basal diet on an as-fed basis^*c*^

Ingredient	% basal diet
Corn	50.06
Dehulled soybean meal	26.76
Whey powder	10
Soy protein (HP300)	7.5
Pork fat (choice white grease)	2
Calcium phosphate	1.05
Limestone, ground	1.1
Sodium chloride	0.3
l-Lysine hydrochloride	0.3
Vitamin premix without phytase^*a*^	0.25
Zinc oxide	0.25
dl-Methionine	0.16
l-Threonine	0.11
Trace mineral premix^*b*^	0.15
Feed enzymes (HiPhos 2700)	0.015

a Vitamin premix provided the following per ton of diet: vitamin A, 1 × 10^7^ IU; vitamin D, 1.25 × 10^7^ IU; vitamin E, 4 × 10^4^ IU; vitamin B_12_, 35 mg; niacin, 45,000 mg; pantothenic acid, 25,000 mg; riboflavin, 7,500 mg.

b Trace mineral premix provided the following per ton of diet: zinc, 1,965 ppm; iron, 165 ppm; manganese, 40 ppm; copper, 17 ppm; iodine, 0.30 ppm; selenium, 0.30 ppm.

c This diet delivered 3,381 kcal/kg, 22.7% crude protein, 1.35% standardized ileal digestible lysine, 34% ileal digestible methionine-lysine, 57% ileal digestible methionine and cysteine-lysine, 0.8% calcium, and 0.67% phosphate.

### Animal study design.

Twenty male pigs born to six sows in summer 2019 at the OSU Swine Facility in Dublin, OH, were used in this study. Male pigs were selected to allow sampling of prostatic tissue for a secondary study. At weaning, 20 male pigs were selected according to weight and randomly assigned to dietary treatment. A scheme of the overall study design can be found in Fig. 1.

To prevent diet mixing and cross-contamination of microbiomes through contact, only pigs consuming the same diets were allowed to have contact. The two diet groups were housed across the room from each other and divided by a walkway. Pens had sufficient space between railings for nose-to-nose contact with other pigs, though not enough space to allow a pig to leave its own pen. After successful weaning from mother’s milk, all pigs consumed the basal diet to acclimate to solid food from week 3 to 4. Pigs at 4 weeks of age began consuming the experimental diets assigned. Feeders were attached to the front of the pens and allowed pigs to eat *ad libitum*. Pigs were weighed weekly to monitor growth and were checked daily to ensure health. Apart from feeding, weighing, and swabbing, human contact with pigs was minimized to limit influences on the gut microbiome of pigs. This study was approved by the OSU Office of Responsible Research Practices (IACUC no. 2019A00000060).

### Sample collection.

The microbiome was sampled 3 times during this study via rectal swabs: prior to beginning experimental diets (day 0, aged 4 weeks), after 1 week of consuming assigned diets (day 7, the study midpoint, aged 5 weeks), and after 2 weeks of dietary intervention (day 14, end of study, aged 6 weeks) (Fig. 1). Swabs used for collection were sterile DNA/RNA shield collection tubes (Zymo Research, Irvine, CA, USA) and were stored at −80°C after collection prior to sequencing.

### Sample processing and sequencing.

Swabs were sent to CosmosID, Inc. (Rockville, MD, USA), for DNA extraction and sequencing. Samples were sequenced via 150-bp paired-end shotgun sequencing, using an Illumina HiSeq4000 instrument (San Diego, CA, USA). Unopened collection tubes were used as negative controls. Samples with reads lower than 1.8M reads were resequenced and merged with the prior sequences, allowing increased microbiome coverage.

### Quality of sequences.

Quality of sequences was analyzed using FastQC version 0.11.9 (26). Sequences were trimmed during annotation in MG-RAST version 4.0.3 (68) if they contained more than 5 bases that were below a minimum Phred quality score of 20. Full metadata for MG-RAST parameters can be found at https://www.mg-rast.org/linkin.cgi?project=mgp93233.

### Sequence analyses and taxonomy identification.

Raw fastq files were made publicly available via the NCBI Sequence Read Archive (SRA), project number PRJNA601162. Annotated files are available through MG-RAST (project mgp93233), and annotated taxa can be found in Tables S3 and S4 in the supplemental material. Sample reads were annotated via the MG-RAST open-access pipeline (68) using the RefSeq database (69). No assembly was completed prior to annotation. Sequences were screened for host DNA using the NCBI Sus scrofa v10.2 genome and, if identified, were removed. Sequences from *Bacteria*, *Archaea*, *Eukaryota*, and viruses were kept for further analysis. Phyla and genera were filtered to exclude taxa that were present in less than 67% of tested samples.

### Statistical analysis.

All data analysis was performed in R version 4.0.3 (70) using RStudio (71), and results were considered significant at a *P* value of ≤0.05. All code used to conduct analyses can be found in the tomato-pig-microbiome repository at https://www.github.com/CooperstoneLab. All figures were created using ggplot2 (72). Differences in body weight were tested using a two-tailed *t* test. Microbiome profiles at both the phylum and the genus taxonomic level were analyzed. Data were normalized using relative abundance to account for differences in sequencing depth, since rarefaction is no longer recommended as a normalization tool due to high potential for data loss (73). Relative abundance was calculated by dividing the number of counts for any one taxon by the total number of counts at that taxonomic level per sample. Interactive Krona plots (Fig. S1) were created using R packages phyloseq (74) and psadd (75) to visualize the microbiome composition. To assess sufficiency of sequencing depth, rarefaction curves were created using the package ranacapa (76) with a window size of 60,000 counts (Fig. S1).

To understand overall microbiome differences between diet groups and across time points, beta-diversity was calculated using the R package vegan and functions “vegdist” and “cmdscale” and then visualized using PCoA with a Bray-Curtis dissimilarity matrix. Significance of separation between treatments was tested via restricted permutation tests using permutational multivariate analysis of variance (PERMANOVA) (77) with the R package vegan using the function “adonis2” (78) and the “how” function from the package permute (79) (model: beta-diversity ~ diet + time point + diet × time point + error where each pig was a plot containing 3 samples collected over time). The argument “by” was set to “margin” to assess how much each individual term contributes to the model. The permutations were restricted within each pig as a time series for which the same permutation was used across pigs (R code available in the supplemental material).

To examine differences in relative abundances of individual microorganisms across groups, univariate analyses were conducted using the R package ALDEx2 (45–47). This specific package was used because it is designed to analyze high-throughput sequence data as compositional data (i.e., it accounts for total reads and uses a data transformation for statistical testing), allowing direct comparison of samples without an effect of total number of reads (46, 48). Raw taxon counts (compared to relative abundance data) were used and center log ratio (CLR) transformed for these analyses (45, 46). Parametric tests were used for these analyses as our data met assumptions for normality.

Alpha-diversity of each sample was calculated from counts using the Shannon index in the R package vegan with the function “diversity” (78). The Shannon index alpha-diversity group means were compared using two-way repeated-measures ANOVA (model: alpha-diversity ~ diet + time point + diet × time point + error). *Post hoc* analyses for significant model terms were completed using pairwise comparison via *t* test to determine where differences originated.

The ratio of the phylum *Bacteroidota* (i.e., *Bacteroidetes*) to the phylum *Bacillota* (i.e., *Firmicutes*) was determined for each sample by dividing relative abundance of *Bacteroidota* by that of *Bacillota*, each as a percentage of the total phyla. Differences between the ratios were tested between diets and time points using two-way repeated-measures ANOVA given our *a priori* interest in these phyla, followed by a pairwise comparison via *t* test as a *post hoc* analysis. Additionally, the relative abundances of *Bacteroidota* and *Bacillota* phyla were separately tested using two-way repeated-measures ANOVA with a *post hoc* test of pairwise comparison by *t* test.

### Data availability.

Raw fastq files were made publicly available via the NCBI Sequence Read Archive (SRA), project number PRJNA601162. Annotated files are available through MG-RAST (project mgp93233), and annotated taxa can be found in Tables S3 and S4 in the supplemental material. All code for analysis can be found in the tomato-pig-microbiome repository at https://www.github.com/CooperstoneLab.
