# Supplementary material for: Short-Term Tomato Consumption Alters the Pig Gut Microbiome toward a More Favorable Profile
Source: Microbiol Spectr. 2022 Nov 8;10(6):e02506-22. doi: 10.1128/spectrum.02506-22 (PMC9769997; doi:10.1128/spectrum.02506-22)
Supplement: Supplemental file 2 — Fig. S1. Download spectrum.02506-22-s0002.docx, DOCX file, 0.3 MB [file spectrum.02506-22-s0002.docx]

**SUPPLEMENTAL FIGURE 1**. Rarefaction curves showing species richness relative to sequence sample size, by sampling day and diet.
